# Comparing Capitonnage and Uncapitonnage Techniques for Pulmonary Hydatid Cysts: A Systematic Review and Meta-analysis

**DOI:** 10.5152/eurasianjmed.2023.22281

**Published:** 2023-12-01

**Authors:** Yener Aydın, Kamber Kasalı, Ali Bilal Ulaş, Ayşenur Dostbil, İlker İnce, Atilla Eroğlu

**Affiliations:** 1 Department of Thoracic Surgery, Atatürk University Medical Faculty, Erzurum, Turkey; 2 Department of Anesthesiology, Atatürk University Medical Faculty, Erzurum, Turkey; 3 Department of Biostatistics, Atatürk University Medical Faculty, Erzurum, Turkey; 4 Department of Anesthesiology and Reanimation, Atatürk University Medical Faculty, Erzurum, Turkey

**Keywords:** Hydatid cyst, meta-analysis, capitonnage, uncapitonnage, surgery

## Abstract

Surgery is the primary treatment for pulmonary hydatid cysts. This systematic review and meta-analysis aimed to compare the results of capitonnage and uncapitonnage techniques for the surgery of pulmonary hydatid cysts. Descriptive Boolean queries were used to search PubMed, Scopus, and Web of Science for articles published up to June 2022 to evaluate the outcomes of pulmonary hydatid cysts in terms of mortality, postoperative complications, and hospital stay. A total of 12 studies were included. An analysis of the total side effects revealed that there was a statistically significant difference between the capitonnage and uncapitonnage groups (odds ratio = 3.81, 95% confidence interval = [1.75-8.31], *P* < .001). The results showed that more side effects were observed in the uncapitonnage group than in the capitonnage group. The risk of side effects in the uncapitonnage group is 3.81 times higher than in the capitonnage group. The results showed that more prolonged air leak was seen in uncapitonnage group than in the capitonnage group (odds ratio = 4.18, 95% confidence interval = [1.64-10.64], *P* = .003). The results show that more empyema was observed in uncapitonnage group than in the capitonnage group (odds ratio = 4.76, 95% confidence interval = [1.29-17.57], *P* =0.020). An analysis of atelectasis and mean hospital stay revealed that there was no statistically significant difference between the capitonnage and uncapitonnage groups. The results reveal the advantages of capitonnage in the treatment of pulmonary hydatid cysts and that the capitonnage method is quite effective in reducing complications compared to the uncapitonnage method.

**Main Points**

- In the treatment of pulmonary hydatid cysts, the capitonnage (Cap) method has significant advantages over the uncapitonnage method.
- In the evaluation of all complications, it is significantly less common in the Cap group.
- Prolonged air leak and empyema, which are specific complications, were significantly less common in the Cap group.
- There was no statistically significant difference between the 2 groups when atelectasis and mean hospital stay were compared.
- The Cap method is effective in reducing complications in pulmonary hydatid cyst surgery and does not adversely affect the rate of postoperative atelectasis and hospital stay.

## Introduction

Hydatid cyst disease is a zoonotic disease that is often seen in regions where animal husbandry and agriculture are common and preventive measures are not taken. The lung is the second most frequently involved organ. The right lower lobe of the lung is involved in 36% of the cases, and the left lower lobe in 25%. Bilateral involvement is observed in approximately 14% of cases.^1-4^ Surgery is the gold standard treatment for pulmonary hydatid cysts. Cysts are most often approached with a posterolateral thoracotomy. Some bilateral cysts can be approached with a median sternotomy, but a 2-stage thoracotomy is generally preferred.^5,6^

Various surgical approaches such as resection with enucleation, removal of intact cyst after needle aspiration, pericystectomy, wedge resection, segmentectomy, and lobectomy have been reported in the treatment of pulmonary hydatid cysts.^7-13^

The most frequently applied method among surgical treatment options is the Posadas method, which has been used for about 70 years. The bronchial openings in the pericyst wall are sutured. The cavity walls are approximated either using interrupted purse-string sutures, or the cyst walls are approximated like a “closing book,” often referred to as a capitonnage (Cap). Last, the intact parenchymal ends are approximated using sutures.^9^ On the other hand, it has recently been suggested by some authors that Cap is not necessary, it also inhibits lung expansion, and closure of the bronchial openings is sufficient in all cases.^14,15^ Currently, the most important debate in the surgical treatment of pulmonary hydatid cysts is whether Cap is necessary.^14-25^ This systematic review and meta-analysis aimed to compare the results of Cap and uncapitonnage (Uncap) techniques in the surgery of pulmonary hydatid cysts.

## Materials and Methods

### Search Strategy and Selection Criteria

This systematic review with meta-analysis has been PROSPERO-registered (International Prospective Register of Systematic reviews with the following ID; CRD42022320250) and was reported according to Preferred Reporting Items for Systematic Reviews and Meta-Analyses (PRISMA) guidelines.^26^

Studies comparing the results of Cap and Uncap techniques in pulmonary hydatid cysts written in English up to June 2022 without first date restriction were searched in the PubMed, Web of Science, and Scopus databases. No randomized controlled trials (RCTs) have been seen comparing the 2 techniques. Animal studies, conference summaries, case reports, articles, and reviews not related to pulmonary hydatid cyst surgery were excluded. The keywords used were “Echinococcus” OR “Hydatid” OR “Echinococcosis” OR “Granulosus Infection” AND “Capitonnage.” See the Appendix for a list of search terms and details of study selection.

**Figure 1.**
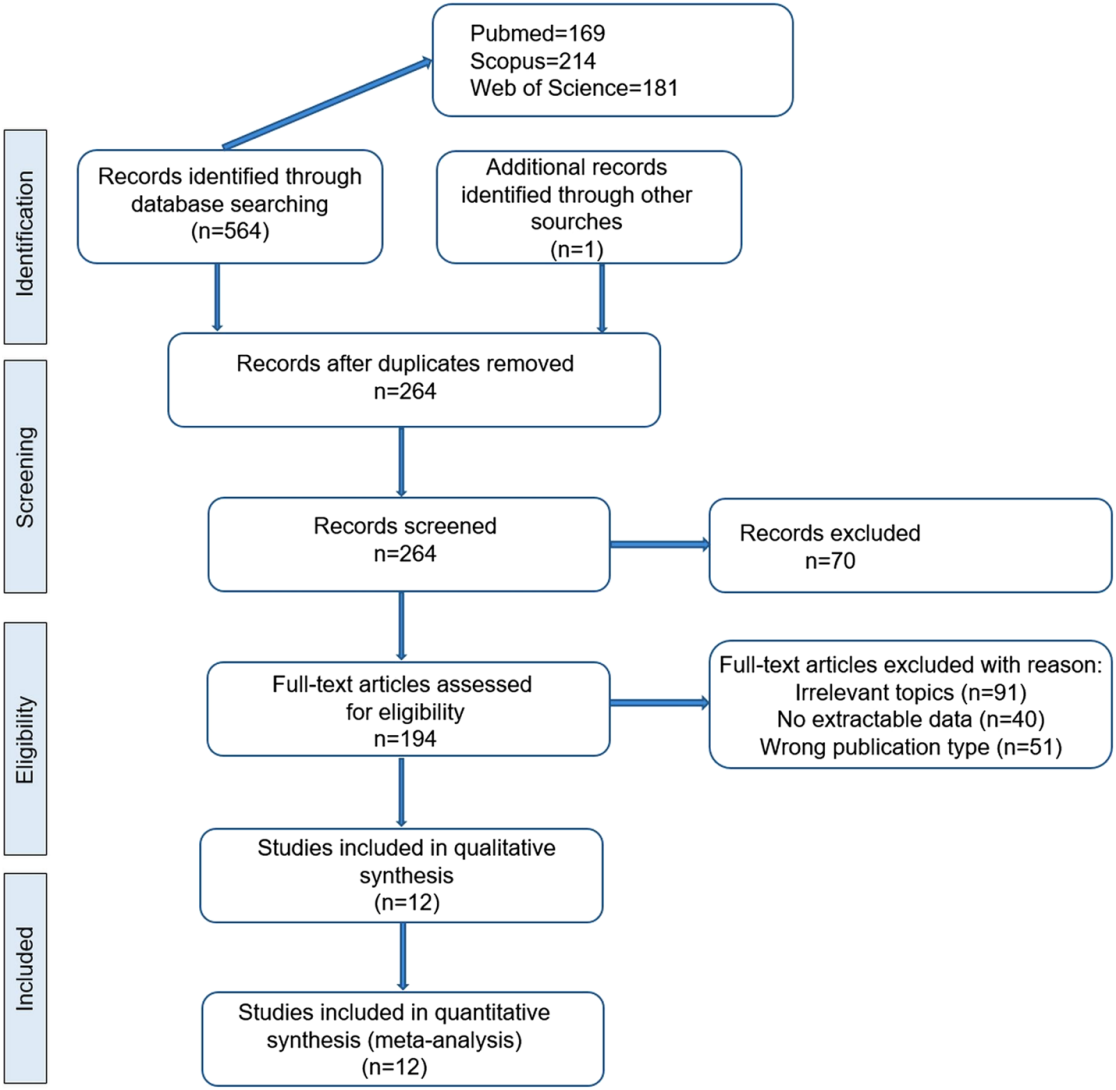
Flow chart of database search.

After the primary election, the articles were scanned first in terms of title and abstract, and then in terms of the full text. Two researchers (Y.A. and A.B.U.) evaluated the studies independently. All disputes regarding the inclusion and exclusion of articles were resolved unanimously. The complete selection process is shown in a PRISMA flowchart (Figure 1). Data extraction was reported per PRISMA guidelines. The primary evaluation results were to determine the overall complication rates for both techniques. Secondary outcomes were a comparison of specific complications such as atelectasis, prolonged air leak, empyema, and mean hospital stay.

The following data was recorded; study style, year of publication, first author, type of intervention, number of patients, study population overall complication rates, specific complications reported such as atelectasis, prolonged air leak, bronchopleural fistula, empyema, mean hospital stay, chest tube removal time, duration of follow-up, recurrence, mortality rates, and conclusions. An overview of the characteristics is shown in Table 1. All comparable data from the studies were included in our analysis.

All 12 studies included in the meta-analysis were retrospective. Since none of the studies was randomized controlled clinical trials, bias (the Cochrane RoB) was not evaluated.^27^

**Figure 2.**
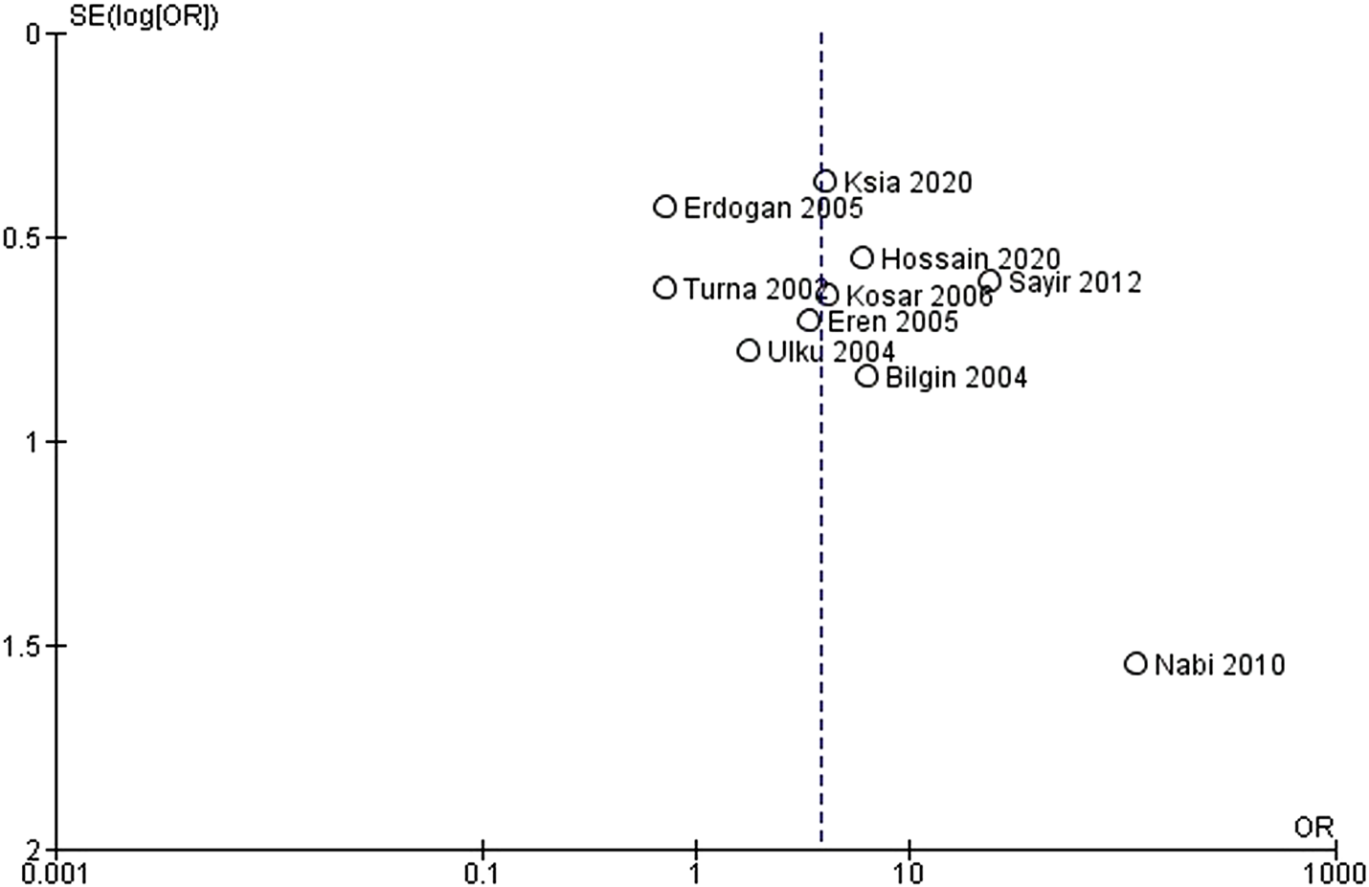
Funnel plot.

All systematic reviews have no publication bias. We analyzed publication bias with funnel plots (Figure 2). This tool is strong enough to determine the publication bias.

### Data Analysis

#### Statistical Methods of Meta-Analysis

The summary data were presented as mean, standard deviation, number of samples, number of events, odds ratio (OR), mean difference (MD), and 95% confidence intervals (CI). For quantitative data (mean hospital stay), we estimated the combined MD with 95% CI. For qualitative data (total side effects, atelectasis, prolonged air leak, and empyema), we estimated the combined OR with 95% CI. Heterogeneity was assessed by the Higgins *I* ^2^ test and chi-square test. When *I* ^2^ ≤ 50%, *P *≥ .10, and the fixed-effect model was applied; otherwise, the random-effect model was used to explore heterogeneity. All results were presented with a forest plot. We did not assess publication bias if the number of studies included was insufficient. We considered *P*-value < 0.05 as statistically significant and preferred 95% CI between studies.

We have prepared our meta-analysis as outlined in thePRISMA guidelines. The meta-analysis was performed using Review Manager Software (Review Manager, version 5.4.1 for Windows; the Nordic Cochrane Centre, Copenhagen, Denmark).

## Results

### Comparison of All Surgery-Related Complications Between Capitonnage and Uncapitonnage

**Figure 3.**
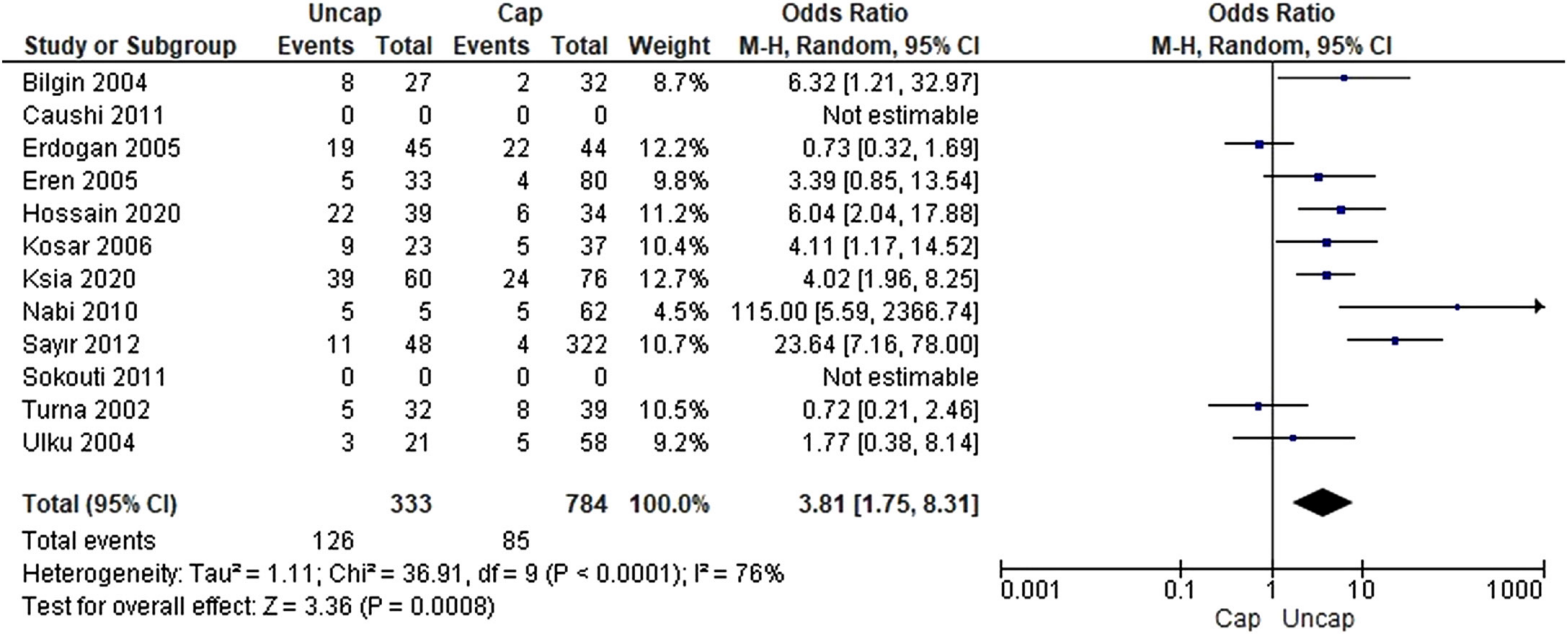
Comparison of all complications.

In the studies, total complication values were calculated between the 2 groups divided as Cap and Uncap. Because of the heterogeneity in the data, the random-effects model was used; (Tau^2^ = 1.11; Chi^2^ = 36.91, *df* = 9; *P *< .001, *I* ^2^ = 76%). An analysis of the side effects showed that there was a statistically significant difference between Cap and Uncap groups (OR = 3.81, 95% CI = [1.75-8.31], *P *< .001. The results show that more side effects were seen in the Uncap group than in the Cap group. The risk of side effects in the Uncap group is 3.81 times higher than in the Cap group. This risk ranges from 1.75 to 8.31 times (Figure 3).

### Comparison of Atelectasis Between Capitonnage and Uncapitonnage

In the studies, atelectasis values were calculated between the Cap and Uncap groups. Because of the heterogeneity in the data, the random-effects model was used; (Tau^2^ = 0.56; Chi^2^ = 16.98, *df* = 8; *P *= .03, *I* ^2^ = 53%). An analysis of the atelectasis showed that there was no statistically significant difference between the Cap and Uncap groups (OR = 0.95, 95% CI = [0.45-2.00], *P *= .90. 

### Comparison of Prolonged Air Leak Between Capitonnage and Uncapitonnage

**Figure 4.**
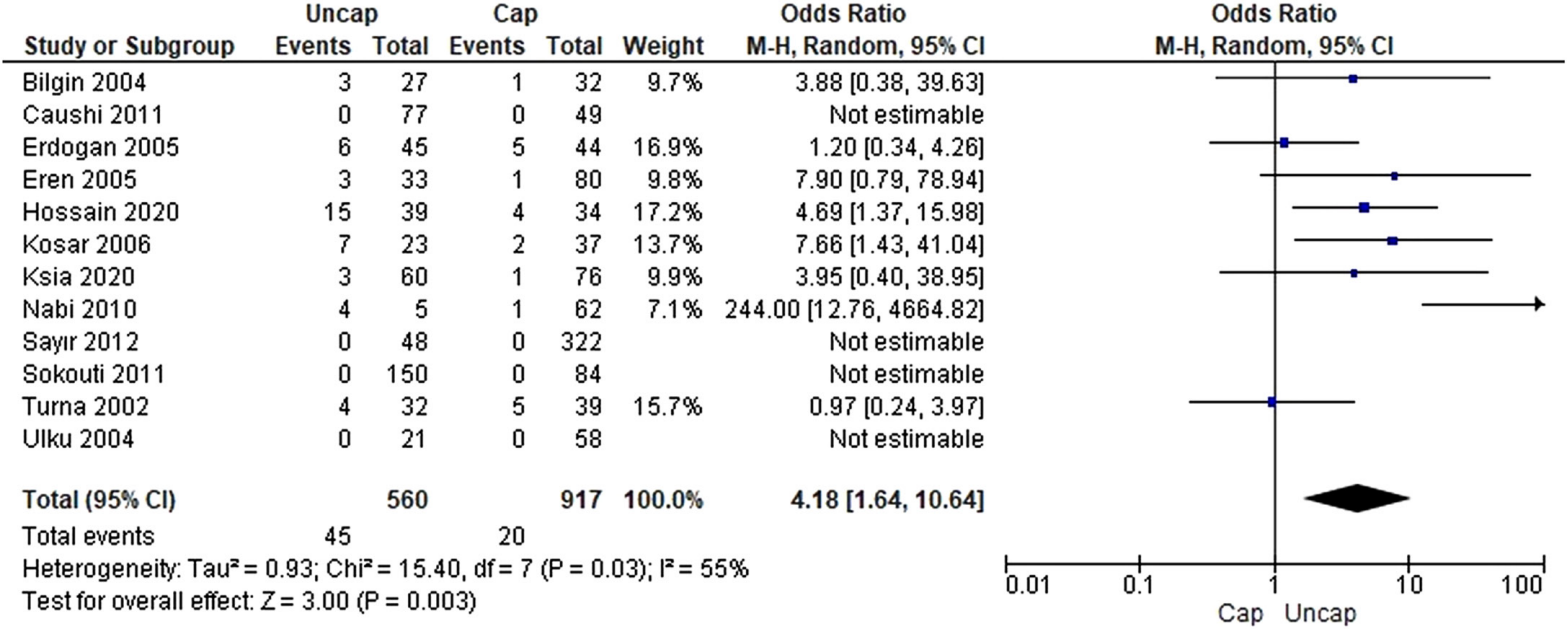
Comparison of prolonged air leak.

In the studies, prolonged air leak values were calculated between the Cap and Uncap groups. Because of the heterogeneity in the data, the random-effects model was used; (Tau^2^ = 0.93; Chi^2^ = 15.40, *df* = 7; *P *= .03, *I* ^2^ = 55%). An analysis of the prolonged air leak showed that there was a statistically significant difference between the Cap and Uncap groups (OR = 4.18, 95% CI = [1.64-10.64], *P *= .003). The results show that more prolonged air leak was seen in the Uncap group than in the Cap group. The risk of prolonged air leak in the Uncap group is 4.18 times higher than in the Cap group. This risk ranges from 1.64 to 10.64 times (Figure 4).

### Comparison of Empyema Between Capitonnage and Uncapitonnage

**Figure 5.**
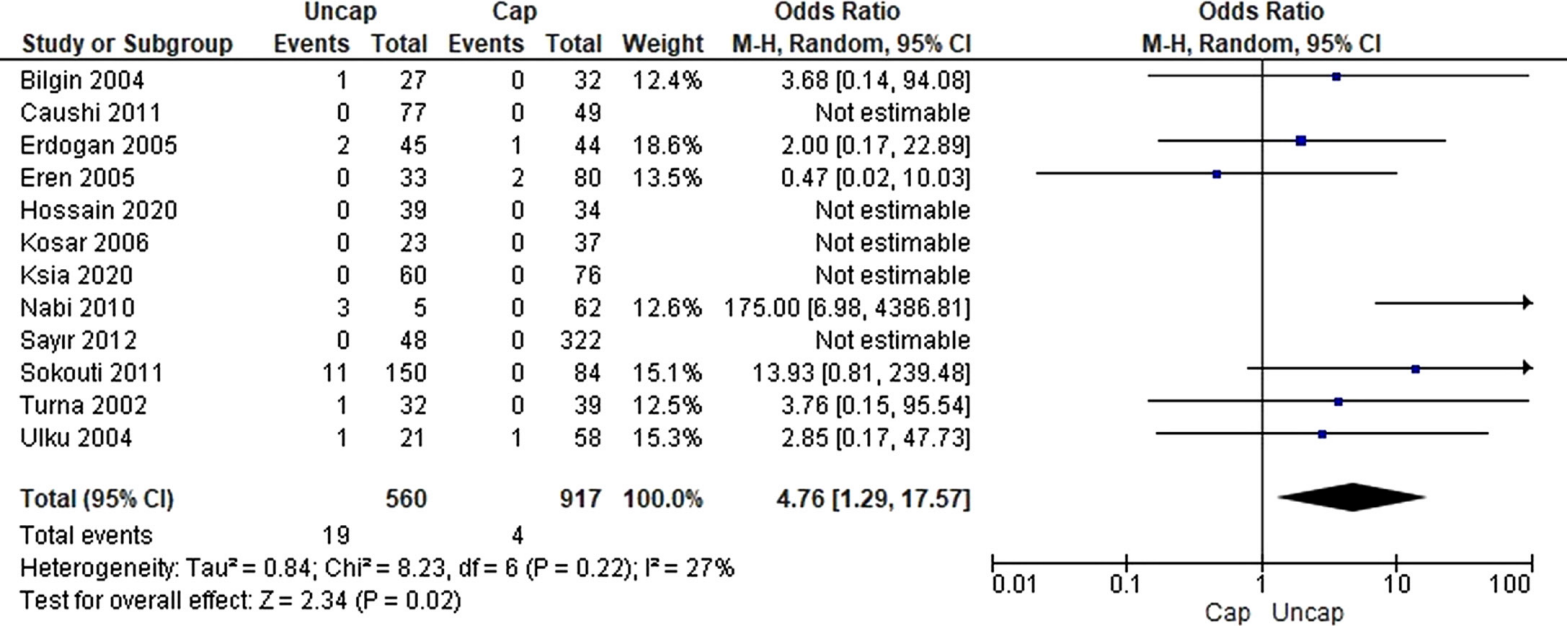
Comparison of empyema.

In the studies, empyema values were calculated between the Cap and Uncap groups. Because of the heterogeneity in the data, the random-effects model was used; (Tau^2^ = 0.84; Chi^2^ = 8.23, *df* = 6; *P *= .22, *I* ^2^ = 27%). An analysis of the empyema showed that there was a statistically significant difference between the Cap and Uncap groups (OR = 4.76, 95% CI = [1.29-17.57], *P=0.020*1. The results show that more empyema was seen in the Uncap group than in the Cap group. The risk of empyema in the Uncap group is 4.76 times higher than in the Cap group. This risk ranges from 1.29 to 17.57 times (Figure 5).

### Comparison of Other Surgery-Related Complications and Mean Hospital Stay Between Capitonnage and Uncapitonnage

The results of the bronchopleural fistula were presented in 2 articles. No statistically significant difference was found between the Cap and Uncap groups. OR = 7.55, 95% CI = [0.67-84.56], *P* > .05.

Wound infection results were presented in 2 articles. No statistically significant difference was found between the Cap and Uncap groups. OR = 3.45, 95% CI = [0.63-18.91], *P* > .05.

The results of pneumothorax–emphysema–pseudocystic appearance were presented in 3 articles. Pneumothorax–emphysema–pseudocystic appearance was statistically less common in the Cap group than in the Uncap group. OR = 2.93, 95% CI = [1.65-5.17], *P* < .001.

Residual cavity results were presented in 4 articles. No statistically significant difference was found between the Cap and Uncap groups. OR = 0.81, 95% CI = [0.27-2.42], *P* > .05.

Pneumonia results were presented in 2 articles. No statistically significant difference was found between the Cap and Uncap groups. OR = 0.47, 95% CI = [0.19-1.17], *P* > .05.

Hemorrhage results were presented in 2 articles. No statistically significant difference was found the between Cap and Uncap groups. OR = 0.53, 95% CI = [0.05-5.23], *P* > .05.

Re-operation results were presented in 4 articles. No statistically significant difference was found between the Cap and Uncap groups. OR = 2.97, 95% CI = [0.37-23.68], *P* > .05.

Recurrence results were presented in 5 articles. No statistically significant difference was found between the Cap and Uncap groups. OR = 1.12, 95% CI = [0.46-2.76], *P* > .05.

The air leak results were presented in 4 articles. No statistically significant difference was found between the Cap and Uncap groups. MD = −1.11, 95% CI = [−4.01-1.80], *P* > .05.

The chest tube removal time results were presented in 3 articles. No statistically significant difference was found between the Cap and Uncap groups. MD = −1.19, 95% CI = [−3.86-1.48], *P* > .05.

In the studies, mean hospital stay values were calculated between the Cap and Uncap groups. Because of the heterogeneity in the data, the random-effects model was used; (Tau^2^ = 7.47; Chi^2^ = 124.17, *df* = 7; *P *< .001, *I* ^2^ = 94%). An analysis of the mean hospital stay showed that there was no statistically significant difference between the Cap and Uncap groups (MD =(−0.22), 95% CI = [(−2.20–(1.76)], *P *= .83. 

## Discussion

The remaining 12 studies containing appropriate and sufficient data for inclusion in the current meta-analysis were used. In this meta-analysis comparing the results of Cap and Uncap techniques in pulmonary hydatid cyst surgery, it was found that complications were generally 3.81 times less common in the Cap group than in the Uncap group. Prolonged air leak and empyema, which are specific complications, were found to be 4.18 and 4.76 times less frequent in the Cap group than in the Uncap group, respectively. There was no significant difference between the 2 groups in the comparison of atelectasis and mean hospital stay. Recurrence rates were presented in 5 articles, air leak, residual cavity, re-operation rates in 4 articles, chest tube removal time, pneumothorax–emphysema–pseudocystic appearance rates in 3 articles, and bronchopleural fistula, hemorrhage, pneumonia, wound infection rates in 2 articles. Although data on these complications are scarce, no statistically significant difference was found between the Cap and Uncap groups. It is obvious that the Uncap approach has a shorter operative time. However, we see that the duration of surgery was not compared in the 12 studies examined.

Although there are some different approaches among surgeons, surgical treatment, which is accepted as the primary treatment of pulmonary hydatid cysts, has been applied all over the world for a long time.^7,28-31^ To date, different surgical approaches have been applied to remove the cyst, repair the lung, and minimize the possible complications and recurrence risks. Considering the location of the lesion and ease of access, thoracotomy incisions of varying sizes and even video-assisted thoracoscopic surgery or hybrid interventions are performed, although not as much as open surgery.^32-35^ The surgical treatment aims to remove the endocyst together with the daughter vesicles while preserving as much lung tissue as possible, to prevent the rupture of the cyst at the operation site, and close the bronchial openings in the cyst wall if any.^10,11^ The most important debate in the surgery of pulmonary cysts is whether obliteration (Cap) is required following cystotomy.^14-25^ In most studies, Cap is generally advocated for the obliteration of residual space.^17-20^ However, especially in the 2000s, studies have been published that argue that Cap is not necessary and that only closure of bronchial leaks will heal the expandable parenchyma.^14,15^ Although there are many studies in the literature on the surgical treatment of pulmonary hydatid cysts, comparative studies between these 2 procedures are limited and there is no meta-analysis study comparing the 2 techniques in the English literature so far. The studies in the literature comparing Cap and Uncap treatment techniques for pulmonary hydatid cysts are all retrospective and there are no prospective RCTs.

The Cap process is used to obliterate the residual space and also to prevent postoperative air leak and empyema formation.^9^ The authors defending the Uncap technique, on the other hand, state the disadvantage of distorting the pulmonary parenchyma, especially after the removal of multiple cysts and closure of the mouths of the major bronchi. It is reported that this situation will result in atelectasis by restricting the re-expansion of the lung after surgery.^9^ However, there are also studies stating that atelectasis is not seen or seen at a very low rate after the Cap procedure.^13,18^ In addition, another important issue is to reduce hospital costs by shortening the hospitalization period in patients who do not undergo Cap.^14^ However, in some studies, it has been emphasized that the mean hospital stay is longer and the complication rates are markedly higher in cases without Cap and that Cap is a necessary procedure.^18,22^ The current meta-analysis shows that there is no statistically remarkable difference in terms of both atelectasis and length of hospital stay. This situation appears to occur due to increased complications in Uncap cases. In addition, these increased complication rates negatively affect the cost.

As noted in most studies, the most common complication in both the Cap and Uncap groups was prolonged air leak.^18^ Prolonged air leakage is important morbidity after hydatid cyst surgery and it was detected 4.18 times more in the Uncap group than in the Cap group in the current meta-analysis. After closing the obvious bronchial openings, small bronchial openings can also be easily detected by filling the residual cavity with normal serum solution. Using positive pulmonary pressure, leakage from any bronchial opening can be visible by the formation of air bubbles. Unnoticed bronchial openings due to visual obstruction caused by blood clots and secretions likely cause a higher incidence of air leakage in cases without Cap.

One of the concerns of the authors advocating the Uncap technique is that the Cap sutures cause laceration and infection in the lung tissue, especially in infected, giant, and complicated cysts.^16,36^ Although this is an important concern, effective techniques can be used to secure the lung parenchyma and sutures by modifying the Cap method in such cases.^1^

### Limitations

Even though this study comprehensively evaluated and commented on the efficacy of Cap and Uncap techniques in the surgical treatment of pulmonary hydatid cysts, there are a few limitations listed as follows:

1. Due to the lack of randomized controlled studies on the surgical treatment of pulmonary hydatid cysts, they were not included in our study. Therefore, 12 nonrandomized articles were included in the study.
2. Because non-English studies were not included, there may be minor influences on the results of our analysis.
3. In meta-analysis studies, the role of sample size is very important. In one of the included articles, there were 5 cases in the Uncap group. In addition, in a study, giving only general complication rates and not sharing data on specific complications may also reduce the strength of the statistical meta-analysis approach.
4. Although chest tube removal time, air leak duration, mean intensive care unit stay, and hospital costs are important data in these cases, the inability to obtain sufficient data on these parameters from the studies may limit the scope of meta-analysis.

## Conclusions

In this study, a systematic review and meta-analysis were made on the articles published in English in the literature, and the results reveal the important advantages of the Cap method over the uncap method in the treatment of pulmonary hydatid cysts. In general, it was determined that complications were significantly less common in the Cap group, and similarly, prolonged air leak and empyema, which are specific complications, were significantly less common in the Cap group. It was observed that the Uncap method, which was especially advocated for the reduction of postoperative atelectasis, did not make a statistical difference in the rates of atelectasis development. In addition, when the mean hospital stay times between the 2 groups were compared, it was seen that there was no statistically significant difference. The present meta-analysis and review show that the Cap method is effective in reducing complications in pulmonary hydatid cyst surgery and does not adversely affect the rate of postoperative atelectasis and hospital stay.

## Figures and Tables

**Table 1.** Characteristics of the Included Studies

Author and Publication Year	Design	Average Age/Male to Female Ratio/Surgical Technique/Cyst Size (cm) (Capitonnage/Uncapitonnage)	Mean Hospital Stay (Days)/Chest Tube Removal Time (Days)	Duration of Follow-Up	Outcome Death/Recurrence	Complications	Variable Used for Prognostic/Conclusion
Nabi et al (2010)^23^	Retro	23.5 Cap M/F = none Uncap M/F = none (Cap and Uncap = 62/5)	None	2 months-7 years	Cap (0/0) Uncap (0/0)	Cap (5/62) Uncap (5/5) Atelectasis (Cap = 3 Uncap = 1) Prolonged air leak (Cap = 1 Uncap = 4) Bronchopleural fistula (Cap = 0 Uncap = 1) Empyema (Cap = 0 Uncap = 3) Wound infection (Cap = 1 Uncap=2) Re-operation (Cap = 0 Uncap = 0) Mean complication rate (mean; 95% confidence interval) (Cap = 0.12% (0.08; 0.151) Uncap = 44% (2.20; 3.81)	Mortality. Recurrence. Complications. Cap with cystotomy may be a preferred procedure due to its lower rate of complications.
Ksia et al (2020)^17^	Retro	Cap = 7.8 Uncap = 8.1 Cap M/F = 41/35 Uncap M/F = 30/30 (Cap and Uncap = 76/60) Cap = 7.02 Uncap = 7.7	Cap = 5.68 Uncap = 6.72 Cap = 4.0 Uncap = 4.9	40 months	Cap (0/0) Uncap (0/1)	Cap (24/76) Uncap (39/60) Pneumothorax + emphysema (Cap = 10 Uncap=18) *P* = .028 Atelectasis (Cap = 11 Uncap = 6) Residual cavity (Cap = 7 Uncap = 16) Prolonged air leak (Cap = 1 Uncap = 3) Re-operation (Cap = 0 Uncap = 0)	Mortality. Recurrence. Complications. Cap appears to significantly prevent pneumothorax, residual cavity, and emphysema formation in the long term. Capitonnage prevents hospitalizations and prolonged postoperative air leaks by a very small margin.
Sokouti et al (2011)^16^	Retro	Cap = 28.81 ± 9.37 Uncap = 31.04 ± 8.62 Cap M/F = 40/44 Uncap M/F = 72/78 (Cap and Uncap = 84/150)	Cap = 5.2 ± 2.3 Uncap = 5.9 ± 3.1 (*P* = .03)	2 years	Cap (0/1) Uncap (0/3) (*P* = .54)	Cap (52/84) Uncap (86/150) Minor air leak and pneumothorax (Cap = 9 (10.7%) Uncap = 38 (25.3%) (*P* < .001) Duration of air leak (days) (Cap = 2.1 ± 1.8 Uncap = 6.7 ± 3.5 (*P* < .001) Atelectasis (Cap = 40 (48%) Uncap = 31 (21%) Empyema (Cap = 0 (0%) Uncap = 11 (7.4%) (*P* < .001) Pneumonia (Cap = 3 Uncap = 6) Re-operation (Cap = 0 Uncap = 31) (*P* < .001)	Mortality. Recurrence. Complications. The Cap series had surprisingly higher and better results than the Uncap approaches. For uncomplicated pulmonary hydatid cysts, the Cap approach is superior to the Uncap method as it reduces complications, morbidity, and reoperation rate.
Caushi et al (2011)^20^	Retro	23.5 Cap M/F = none Uncap M/F = none (Cap and Uncap = 49/77)	Cap = 11.38 ± 4.6 Uncap = 3.9 ± 7.5	6 months-9 years	Cap (0/0) Uncap (0/0)	Duration of air leak (days) (Cap = 4.51 ± 2.888 Uncap = 4.42 ± 2.117	Mortality. Recurrence. Complications. Since the use of Cap does not provide any advantage in terms of hospitalization period, it cannot be said to be the best option in the surgical treatment of pulmonary echinococcosis.
Hossain et al (2020)^22^	Retro	Cap = 30.11 ± 11.43 Uncap = 31.05 ± 11.76 Cap M/F = 23/11 Uncap M/F = 25/14 (Cap and Uncap = 34/39)	Cap = 7.5 ± 2.67 Uncap = 10.03 ± 4.53 (*P* = .0476) Cap = 3.41 ± 2.60 Uncap = 5.9 ± 3.2 (*P* = .00084)	1 year	Cap (0/0) Uncap (0/0)	Cap (6/34) Uncap (22/39) Prolonged air leak (Cap = 4 Uncap = 15) Atelectasis (Cap = 2 Uncap = 6) Re-operation (Cap = 0 Uncap = 1) (*P* = .008214)	Mortality. Recurrence. Complications. Both lung-sparing surgical techniques for pulmonary hydatid disease are effective without recurrence and mortality of the hydatid cyst. However, the combined application of cystotomy, closure of the bronchial openings, and Cap provide better outcomes in terms of postoperative air leakage, hospital stay, and re-intervention rates compared to cystotomy alone.
Turna et al (2002)^15^	Retro	Cap = 25.8 ± 17.6 Uncap = 34.3 ± 15.9 Cap M/F = 21/18 Uncap M/F = 20/12 (Cap and Uncap = 39/32) Cap = 6.2 ± 3.6 Uncap = 6.7 ± 3.9	Cap = 5.0 ± 5.0 Uncap = 5.9 ± 6.9	22 months	Cap (0/3) Uncap (0/0)	Cap (8/39) Uncap (5/32) Duration of air leak (days) (Cap = 2.56 ± 4.73 Uncap = 2.38 ± 4.74 Atelectasis (Cap = 0 Uncap = 0) Empyema (Cap = 0 Uncap = 1) Prolonged air leak (>7 days) (Cap = 5 Uncap = 4) Re-operation (Cap = 0 Uncap = 0)	Mortality. Recurrence. Complications. Cap did not provide an advantage in the surgical intervention of lung hydatid cysts in the context of short- and long-term surgical complications and postoperative variables. While closing the bronchial openings is of great importance, Cap may not be performed to shorten the operation time.
Bilgin et al (2004)^19^	Retro	Cap = 23 ± 14.8 Uncap = 27 ± 15.4 Cap M/F = 26/6 Uncap M/F = 22/5 (Cap and Uncap = 32/27)	Cap = 9.8 ± 2.1 Uncap = 12.4 ± 3.2 *P* < .01	30.2 months	Cap (0/0) Uncap (0/0)	Cap (2/32) Uncap (8/27) *P* < .01 Atelectasis (Cap = 1 Uncap = 2) Empyema (Cap = 0 Uncap = 1) Prolonged air leak (>5 days) (Cap = 1 Uncap = 3) Pseudocystic appearance (Cap = 0 Uncap = 2) Re-operation (Cap = 0 Uncap = 0)	Mortality. Recurrence. Complications. Cap is a technique that should not be abandoned easily due to its low complication rate despite current contrary opinions.
Eren et al (2005)^14^	Retro	Cap = 30.1 ± 18.2 Uncap = 27.6 ± 11.4 (Cap and Uncap = 80/33) Cap = 7.4 ± 3.7 Uncap = 6.6 ± 3.4	Cap = 11.7 ± 3.15 Uncap = 8.35 ± 2.3 (*P* < .05) Cap = 4 ± 1.5 Uncap = 3 ± 0.2	51.9 months (range, 11-88 months).	Cap (0/0) Uncap (0/0)	Cap (4/80) Uncap (5/33) *P* < .05 Atelectasis (Cap = 1 Uncap = 2) Empyema (Cap = 2 Uncap = 0) Prolonged air leak (>10 days) (Cap = 1 Uncap = 3) Re-operation (Cap = 2 Uncap = 0)	Mortality. Recurrence. Complications. The Cap procedure is not essential in the surgical treatment of pulmonary hydatid cysts. Careful closure of bronchial openings reduces morbidity.
Kosar et al (2006)^18^	Retro	12.2 Cap M/F = none Uncap M/F = none (Cap and Uncap = 37/23)	Cap = 4.86 ± 1.34 Uncap = 7.22 ± 3.34 (*P* = .003) Cap = 3.59 ± 1.04 Uncap = 5.83 ± 2.84 (*P* = .001)	56 months (13-86 months)	Cap (0/2) Uncap (0/1)	Cap (5/37) Uncap (9/23) (*P* = .031) Atelectasis (Cap = 1 Uncap = 0) Prolonged air leak (>7 days) (Cap = 2 Uncap = 7 *P* = .004) Residual pleural space (Cap = 0 Uncap = 2) Pneumonia (Cap = 1 Uncap = 0) Wound infection (Cap = 1 Uncap = 0) Re-operation (Cap = 0 Uncap = 0)	Mortality. Recurrence. Complications. Cap is a safer technique with lower complication rates (especially for long-term air leakage) than cystotomy alone.
Erdogan et al (2005)^21^	Retro	Cap = 33.2 ± 10.9 Uncap = 35.5 ± 14.4 Cap M/F = 25/19 Uncap M/F = 24/21 (Cap and Uncap = 44/45) Cap = 9.3 ± 3.4 Uncap = 8.4 ± 4.2	Cap = 8.23 ± 3.2 Uncap = 8 ± 3.1	1 year	Cap (0/3) Uncap (0/5)	Cap (22/44) Uncap (19/45) Duration of air leak (days) (Cap = 4.2 ± 3 Uncap = 4.1 ± 2.3) Atelectasis (Cap = 13 Uncap = 9) Empyema (Cap = 1 Uncap = 2) Prolonged air leak (>7 days) (Cap = 5 Uncap = 6) Pneumonia (Cap = 2 Uncap = 1) Bronchopleural fistula (Cap = 0 Uncap = 1) Hemorrhage (require reexploration) (Cap = 1 Uncap = 0) Re-operation (Cap = 2 Uncap = 3)	Mortality. Recurrence. Complications. Whether the cysts are intact or complicated, both techniques give good results in lung hydatid cyst surgery. However, Cap does not provide any advantage in terms of postoperative variables and short- and long-term surgical complications. It is very important to close the bronchial openings. There is no need for Cap after the bronchial openings are closed.
Sayir et al (2012)^24^	Retro	23.6 Cap M/F = none Uncap M/F = none (Cap and Uncap = 322/48)	Cap = 7.8 Uncap = 13.6 (days) (*P* = .001)	3.8 years (3 months-9 years)	Cap (0/none) Uncap (0/none)	Cap (4/322) Uncap (11/48) 0.041 (*P* = .041)	Mortality. Recurrence. Complications. Cap should always be performed in the surgical treatment of hydatid cyst
Ulku et al (2004)^25^	Retro	9.6 ± 7 Cap M/F = none Uncap M/F = none (Cap and Uncap = 58/21)	None	6 months-2 years	Cap (0/0) Uncap (0/0)	Cap (5/58) Uncap (3/21) Atelectasis (Cap = 2 Uncap = 1) Empyema (Cap = 1 Uncap = 1) Pneumonia (Cap = 0 Uncap = 1) Wound infection (Cap = 1 Uncap = 0) Hemorrhage (Cap = 1 Uncap = 0) Re-operation (Cap = 0 Uncap = 0)	Mortality. Recurrence. Complications.

Cap, capitonnage; Uncap, uncapitonnage.
